# Diffusion Weighted Imaging in Gliomas: A Histogram-Based Approach for Tumor Characterization

**DOI:** 10.3390/cancers14143393

**Published:** 2022-07-13

**Authors:** Georg Gihr, Diana Horvath-Rizea, Patricia Kohlhof-Meinecke, Oliver Ganslandt, Hans Henkes, Wolfgang Härtig, Aneta Donitza, Martin Skalej, Stefan Schob

**Affiliations:** 1 Katharinenhospital Stuttgart, Clinic for Neuroradiology, 70174 Stuttgart, Germany; d.horvath-rizea@klinikum-stuttgart.de (D.H.-R.); h.henkes@klinikum-stuttgart.de (H.H.); 2 Katharinenhospital Stuttgart, Department for Pathology, 70174 Stuttgart, Germany; p.kohlhof@klinikum-stuttgart.de; 3 Katharinenhospital Stuttgart, Clinic for Neurosurgery, 70174 Stuttgart, Germany; o.ganslandt@klinikum-stuttgart.de; 4 Paul Flechsig Institute for Brain Research, University of Leipzig, 04103 Leipzig, Germany; wolfgang.haertig@medizin.uni-leipzig.de; 5 Department for Neuroradiology, Clinic and Policlinic for Radiology, University Hospital Halle (Saale), 06120 Halle (Saale), Germany; aneta.donitza@uk-halle.de (A.D.); martin.skalej@uk-halle.de (M.S.)

**Keywords:** astrocytoma, glioblastoma, LGG, HGG, DWI, histogram, tumor biology, radiomics

## Abstract

**Simple Summary:**

Glioma represent approximately one-third of all brain tumors. Although they differ clinically, histologically and genetically, they often are not distinguishable by morphological magnetic resonance imaging (MRI) diagnostics. We therefore investigated in this retrospective study whether diffusion weighted imaging (DWI) using a radiomic approach could provide complementary information with respect to tumor differentiation and cell proliferation, as well as the underlying genetic and epigenetic tumor profile. We identified several histogram features that could facilitate presurgical tumor grading and potentially enable one to draw conclusions about tumor characteristics on a cellular and subcellular scale.

**Abstract:**

(1) Background: Astrocytic gliomas present overlapping appearances in conventional MRI. Supplementary techniques are necessary to improve preoperative diagnostics. Quantitative DWI via the computation of apparent diffusion coefficient (ADC) histograms has proven valuable for tumor characterization and prognosis in this regard. Thus, this study aimed to investigate (I) the potential of ADC histogram analysis (HA) for distinguishing low-grade gliomas (LGG) and high-grade gliomas (HGG) and (II) whether those parameters are associated with Ki-67 immunolabelling, the isocitrate-dehydrogenase-1 (IDH1) mutation profile and the methylguanine-DNA-methyl-transferase (MGMT) promoter methylation profile; (2) Methods: The ADC-histograms of 82 gliomas were computed. Statistical analysis was performed to elucidate associations between histogram features and WHO grade, Ki-67 immunolabelling, IDH1 and MGMT profile; (3) Results: Minimum, lower percentiles (10th and 25th), median, modus and entropy of the ADC histogram were significantly lower in HGG. Significant differences between IDH1-mutated and IDH1-wildtype gliomas were revealed for maximum, lower percentiles, modus, standard deviation (SD), entropy and skewness. No differences were found concerning the MGMT status. Significant correlations with Ki-67 immunolabelling were demonstrated for minimum, maximum, lower percentiles, median, modus, SD and skewness; (4) Conclusions: ADC HA facilitates non-invasive prediction of the WHO grade, tumor-proliferation rate and clinically significant mutations in case of astrocytic gliomas.

## 1. Introduction

Gliomas, along with rarer tumors arising from neuroepithelial tissue, represent circa 28 percent of all brain tumors [1]. They derive from the enduring glia of the brain and are either grouped as astrocytoma or oligodendroglioma, depending on the glial cell type they originate from. Furthermore, based upon histopathologic tumor characteristics and with increasing importance related to genetic alterations, the World Health Organization (WHO) taxonomy subdivides gliomas from the lowest grade, I, to the highest grade, IV. Whereas WHO grade I and II gliomas, which usually present a rather benign tumor biology, are classified as low-grade gliomas (LGG), entities of WHO categories III and IV, which exhibit a more aggressive tumor behavior, are classified as high-grade gliomas (HGG). With about 14.6 percent of all newly diagnosed brain tumors, WHO grade IV astrocytoma (or glioblastoma, GBM) represents the most frequent entity, followed by WHO grade II astrocytoma (1.8 percent), then WHO grade III astrocytoma (1.7 percent) and WHO grade I astrocytoma (1.3%) [1]. In fact, gliomas are tumors of substantial heterogeneity, both in terms of the underlying histopathology, manifestation age and the associated clinical course. For example, WHO grade IV astrocytoma, being the most aggressive glioma, is associated with survival rates of 15 months on average [2], whereas WHO grade I (pilocytic) astrocytoma, the most frequent pediatric glioma, is associated with a rather uneventful further course [3].

Since the 2016 update of the WHO classification of CNS tumors, the diagnostic paradigm in case of gliomas shifted from a predominantly histological perspective towards a genetic- and molecular-based approach [4]. The classification of diffuse gliomas (WHO II-IV), in particular, now includes mutation analysis of the isocitrate dehydrogenase (IDH) 1 and 2 gene, the telomerase (TERT) promoter, the transcriptional regulator (ATRX) gene and the tumor suppressor gene TP53, as well as methylation analysis of the (MGMT) promoter and detection of 1p/19q co-deletions. Besides the prognostic value of this genetical tumor profile, it is essential for the final diagnosis and supersedes the histological diagnostics. For example, the diagnosis of oligodendroglioma is made only in the presence of a complete 1p/19q co-deletion, regardless of the underlying histological appearance [5].

Magnetic resonance imaging (MRI) is the most important imaging modality for gliomas. Presurgical tumor localization, treatment planning and the visualization of eloquent brain areas are the main tasks of MRI in this context, usually performed via conventional T2- and T1-weighted MRI sequences with 2D or 3D acquisition. However, through partially overlapping tumor morphology and significant interobserver variability, the diagnostic performance with those morphological MRI sequences remains limited for glioma grading, and hence, preoperative risk stratification. To overcome those limitations, advanced MRI approaches, for example MR spectroscopy, DWI and gadolinium-based perfusion techniques have been established and integrated in the majority of clinical MRI protocols. Especially DWI, which provides ADC maps that allow one to quantify the spatial extent of diffusion in vivo on a µm scale [6], enables evaluation of the microscopic architecture of a biological sample [7], which renders the technique an important oncological tool [8]. For example, DWI has been shown to be useful in assessing the growth potential of gliomas [9,10], in glioma grading [11], in estimating the clinical prognosis of glioma patients [12] or even in the differentiation of GBM from brain abscesses [13]. Most DWI studies conducted in the past have focused predominantly on first-order features of the ADC histogram profile, i.e., the mean, median, extreme values and percentiles. However, advanced HA includes an estimation of second-order features, i.e., skewness, entropy and kurtosis of the ADC. The latter allow an assessment of the shape of the ADC value distribution within the lesion and therefore facilitate assessment of tumor heterogeneity [14]. Recent studies have further substantiated the value of advanced HA for tumor grading and evaluation of individual tumor biology based on DWI [10,15,16,17,18,19,20,21] and even signal intensities of conventional T1- and T2-sequences [22,23,24,25].

Nevertheless, despite the recent developments in the field of advanced MRI techniques, there still are no reliable in vivo imaging biomarkers that could lead to an effective reduction in tumor biopsies or even replace them, for example, in the case of suspected low-grade gliomas. Therefore, further imaging studies are necessary to reveal new and to evaluate already known imaging biomarkers.

Considering the available data and the need for improved, non-invasive characterization of gliomas, the aim of this study was to evaluate whether whole tumor HA of ADC profiles has the ability to (i) differentiate LGG from HGG, (ii) predict the proliferative activity of the glioma represented by Ki-67 immunolabelling and (iii) predict the prognostic relevant MGMT (methylguanine-DNA methyl-transferase) MGMT methylation profile, as well as the IDH (isocitrate dehydrogenase) mutation profile.

## 2. Materials and Methods

### 2.1. Patients

Our radiological database was reviewed for patients suffering from primary brain tumors/gliomas. A total of 114 patients who were treated in our center between January 2012 and February 2017 were included. Each diagnosis was confirmed histologically either by biopsy or open tumor surgery. The samples were further processed neuropathologically as outlined below. The patients included had sufficient pre-treatment MRI including DWI and did not exhibit signs of lesional hemorrhage or calcification. Eventually, 82 patients met those criteria. In total, 26 patients had LGG (WHO I: *n* = 7, WHO II: *n* = 19; 12 females, 14 males; average age: 34a) and 56 had HGG (WHO III: *n* = 11, WHO IV: *n* = 45; 22 females, 34 males; average age: 62a). Twenty-three percent (19/82) of the patients revealed an IDH-1 mutation and 71 percent (58/82) an IDH-1 wildtype genotype (for 5 patients no IDH-1 mutation status was available). The methylation of the MGMT promoter was positive in 39 percent (32/82); 43 percent (35/82) had tumors with an unmethylated MGMT promoter. In the remaining 15 patients, no MGMT promoter profile was available. In 9.8 percent (8/82) Ki-67 immunolabelling was not available.

### 2.2. MRI Protocol

MRI was performed in all cases using a 1.5 T MAGNETOM scanner (Aera or Symphony, using a Tx/Rx CP head coil, Siemens, Erlangen, Germany). The scanning protocol consisted of axial T1-weighted (T1w) spin echo (SE) sequences (TR/TE: 453/17, flip angle: 90°, slice thickness: 5 mm, acquisition matrix: 320 × 179, field of view: 230 × 187 mm) before and after the application of a gadolinium-based contrast agent (Gadobutrol, Gadovist, Bayer Schering Pharma, Leverkusen, Germany), an axial T2-weighted (T2w) turbo spin echo (TSE) sequence (TR/TE: 5390/99, flip angle: 150°, slice thickness: 5 mm, acquisition matrix: 512 × 291, field of view: 230 × 187 mm) and an axial DWI sequence (readout-segmented, multi-shot EPI sequence; TR/TE: 5500/103, flip angle 90°, slice thickness: 5 mm, acquisition matrix: 152 × 144, field of view: 230 × 230 mm).

All images were digitalized and reviewed by two experienced readers (DHR, SS) who were blinded regarding the histopathological report. Image analysis was performed using a commercially available PACS workstation (Impax EE R20 XII).

### 2.3. Histogram Analysis of ADC Volumes

T1w images, T2w images and ADC maps were anonymized and extracted from the institutional PACS as DICOM files. Subsequently, histogram analysis was performed via a custom-made, MATLAB-based analysis tool (programmed by N.G. using MATLAB, The Mathworks, Natick, MA, USA) as follows. In the case of contrast-enhancing tumors, T1w (post contrast) images, and in the case of non-enhancing tumors, T2w images, were uploaded to the visual interface displaying the anatomical image in order to correlate and tag the tumor of each patient entirely. Regions of interest (ROIs), which were manually drawn in T1w (post contrast) or T2w images along the corresponding border of the whole visible signal alteration (contrast-enhancing region or T2w hyperintense region) in every slice of detectable tumor, were propagated and co-registered to the corresponding ADC maps. Using the MATLAB library, the following features of the ADC histogram of the whole tumor volume were computed: mean, minimum (min), maximum (max), 10th percentile (p10), 25th percentile (p25), 75th percentile (p75), 90th percentile (p90), modus, median, standard deviation (SD), skewness, kurtosis and entropy.

### 2.4. Immunolabelling and Polymerase Chain Reaction (PCR)—Molecular Neuropathology

Testing was performed as described previously [10]:

Tumor specimens were fixed in formaldehyde and embedded in paraffin for histology, immunolabelling and PCR. The embedded tissue was cut with 3µm thickness and stained with hematoxylin and eosin (H&E). Immunolabelling was done employing antibodies raised against IDH1-R132H (1:20 diluted, cat. no. DIA-H09; Dianova, Hamburg, Germany) and MIB/Ki67 (1:800 diluted; cat. no. M7240; Dako Denmark A/S, Glostrup, Denmark). Histology was digitalized using a Leica microscope equipped with a DFC290 HD digital camera; LAS V4.4 was used for image processing (Leica Microsystems, Wetzlar, Germany). Necrosis and hemorrhage were absent in the investigated samples; the presence of viable tumor cells was also confirmed via microscopy. IDH1 immunolabelling resulting in strong cytoplasmic staining was interpreted as IDH1 positive. The proliferation index was calculated by dividing the Ki67-immunolabelled (stained) cellular nuclei by all nuclei. The area exhibiting the greatest number of Ki67-reactive nuclei was chosen in each sample.

To determine the methylation profile of the MGMT promoter, DNA from each glioma was isolated using 10 µm-thick sections (derived from the paraffin-embedded samples). Extraction was performed with the Maxwell^®^ RSC FFPE Plus DNA Kit AS1720 (Promega, Madison, WI, USA). Unmethylated cytosine residues were converted to uracil by bisulfite treatment using the EpiTect^®^ Bisulfite Kit (QIAGEN, Hilden, Germany). All steps were performed in accordance with the manufacturer’s instructions. PCR with bisulfite-converted DNA was performed for amplification and the methylation profile was evaluated via pyrosequencing using the Therascreen MGMT Pyro^®^ Kit (QIAGEN, in accordance with the manufacturers protocol). A methylation of 10 percent or more was considered methylation positive.

### 2.5. Statistical Analysis

Descriptive statistics, group comparison with significance testing and computation of correlations was performed with GraphPad Prism 8 (GraphPad Software, CA, USA).

The Shapiro–Wilk test was performed to test for Gaussian vs. non-Gaussian distribution. Group comparisons of normally distributed values was performed with Student’s T test; non-normally distributed data were tested employing the Mann–Whitney U Test. Correlations between parameters with normal distribution were computed using Pearson’s Correlation Coefficient. For parameters exhibiting non-Gaussian distribution, the Spearman–Rho coefficient was computed. In all instances, *p*-values < 0.05 were interpreted as statistically significant.

As a last step, aiming to assess the accuracy of the investigated histogram features, the receiver operating characteristics (ROC) curve analysis was calculated, including the area under the curve (AUC). For the estimation of suitable cutoff values, Youden’s index was calculated.

## 3. Results

**Figure 1:**
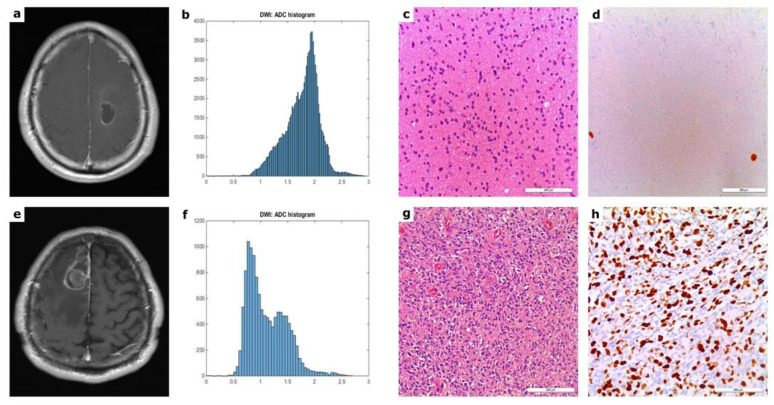
T1w MR images post gadolinium, corresponding ADC histograms, H&E images and Ki67 immunolabelling sections from patients with WHO grade II—(**a**–**d**) and WHO grade IV glioma (**e**–**h**). The upper left image shows a T1w image after intravenous application of a gadolinium-based contrast agent revealing a WHO grade II glioma in the left frontal lobe, extending into the precentral gyrus. The atypical morphologic appearance of this LGG with contrast medium enhancement and cystic areas renders differentiation of this lesion difficult from high-grade neoplasms, based on standard MRI sequences alone (**a**). The lower left image depicts T1w imaging of a WHO grade IV glioma arising from the parafalcine region of the right frontal lobe (**e**). The set of images in the second column show histograms of the respective entire tumor ADC volume (**b**,**f**) The set of images in the third column demonstrate representative H&E stainings (**c**,**g**). The images in the last column (**d**,**h**) demonstrate Ki67 immunolabeling of the lesions. The LGG (WHO grade II) showed a proliferation index of 5%, the HGG had a proliferation index of 50%.

Figure 1 demonstrates the commonly used contrast-enhanced T1-weighted MRI scans of different representative glioma entities (WHO grade II and WHO grade IV), the calculated ADC histogram of the respective tumor, as well as the corresponding histopathological images, consisting of HE staining and Ki-67 immunolabeling.

For better comprehensibility, Table 1 summarizes the results of the descriptive statistical analysis of all included cases.

**Figure 2:**
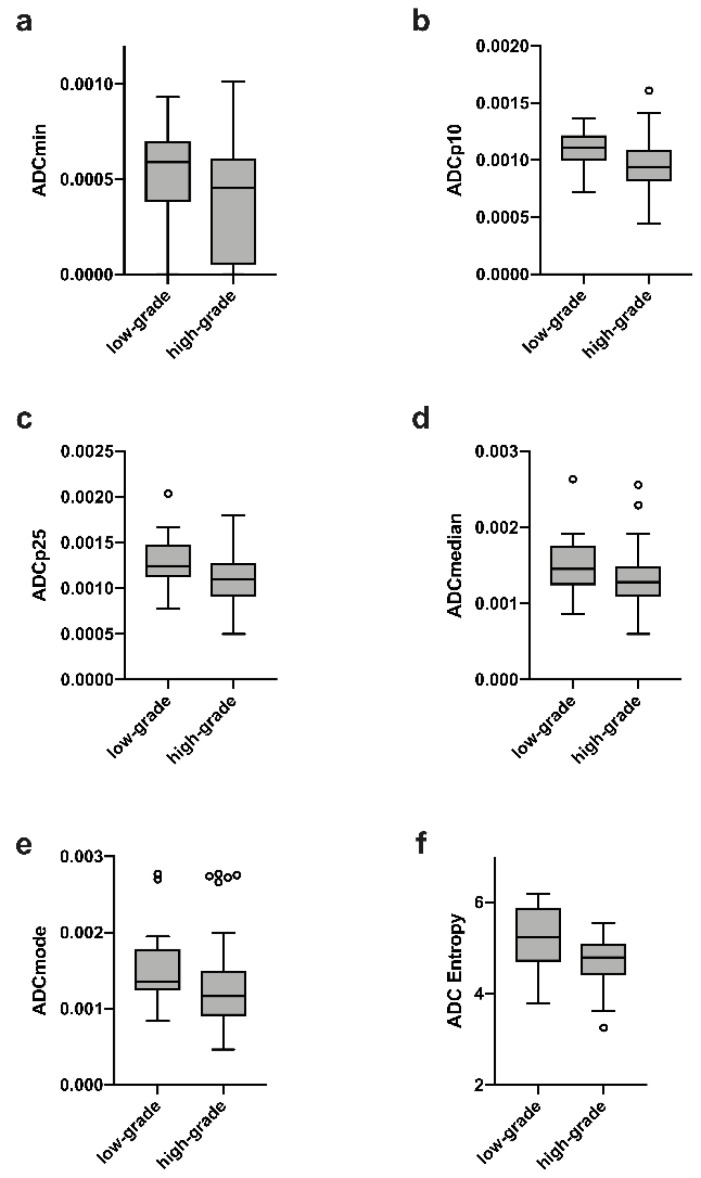
(**a**–**f**) demonstrates significant ADC HA differences between LGG and HGG features (*p* < 0.05).

In brief, statistically significant differences between both entities, LGG vs. HGG, were identified for the following set of ADC features: minimum, lower percentiles (p10 and p25), median, modus and entropy (*p* < 0.05 in all instances). As expected, Ki-67 positive immunolabelling was significantly stronger in HGG compared to LGG. The results of the comparative statistics considering LGG and HGG are listed in Table 2 and graphically demonstrated in Figure 2.

**Figure 3:**
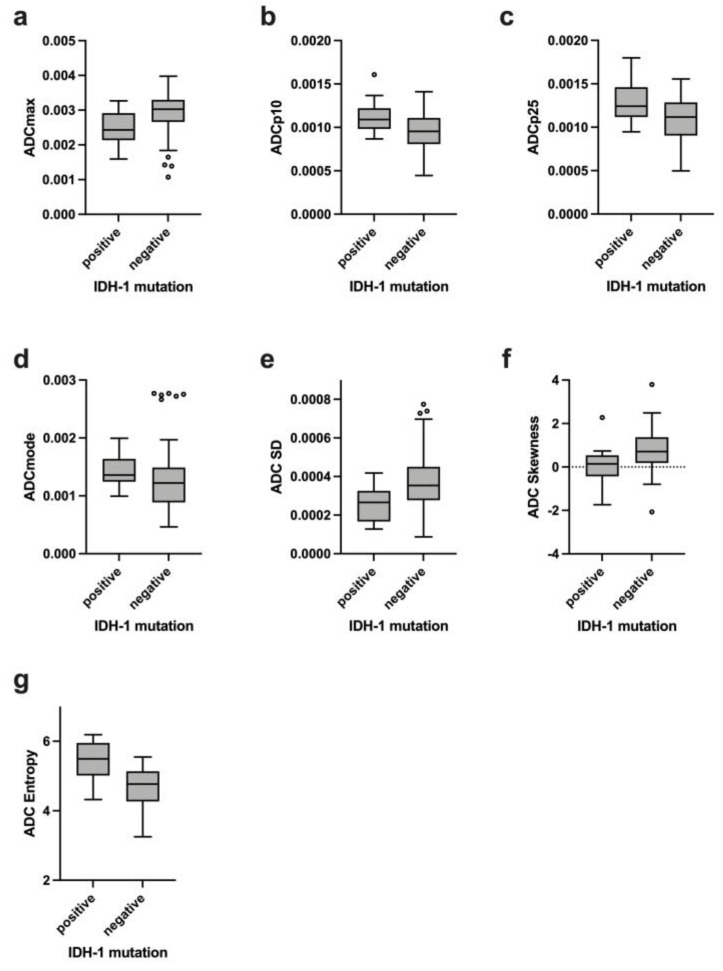
Boxplots (**a**–**g**) demonstrating statistically significant differences of ADC histogram features between IDH-1 mutated and IDH-1 wildtype gliomas (*p* < 0.05).

Comparing IDH1-mutated with IDH1-wildtype gliomas, the following features of the ADC histogram achieved statistical significance: lower percentiles (p10 and p25), modus, standard deviation, maximum, skewness and entropy. The complete results are listed in Table 3 and those with significant differences are shown in Figure 3a–g.

When comparing gliomas with a methylated vs. unmethylated MGMT promoter, no statistically significant differences were detectable, as demonstrated in Table 4.

The computation of correlations between ADC histogram features and Ki67 immunolabelling revealed significant associations (*p* < 0.05) for the following ADC parameters: minimum, lower percentiles (p10 and p25), median, modus, maximum and skewness.

**Figure 4:**
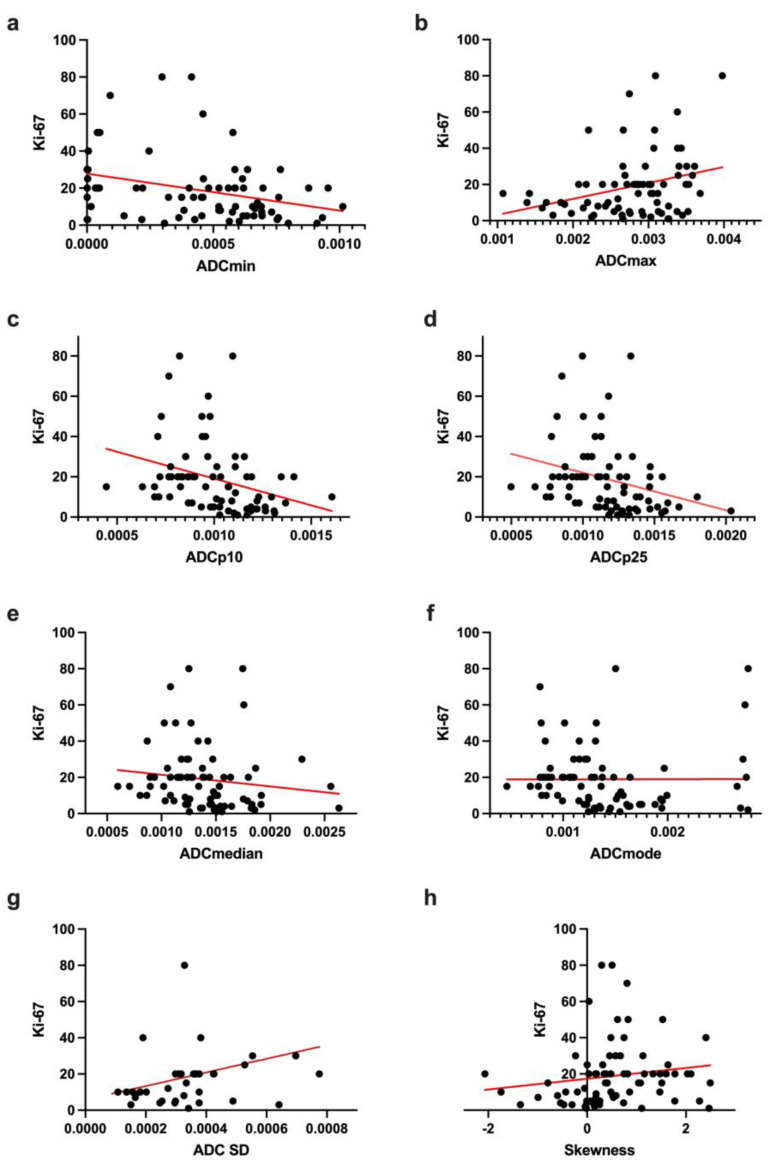
(**a**–**h**) displays the correlations between ADC histogram features (*X*-axis) and the corresponding proliferation index Ki-67 (*Y*-axis) as well as the respective simple linear regression (red line) of all significant correlations.

Table 5 provides all results of the correlative analysis; the strongest correlation was detected for ADCp10. All significant correlations are graphically demonstrated in Figure 4.

**Figure 5:**
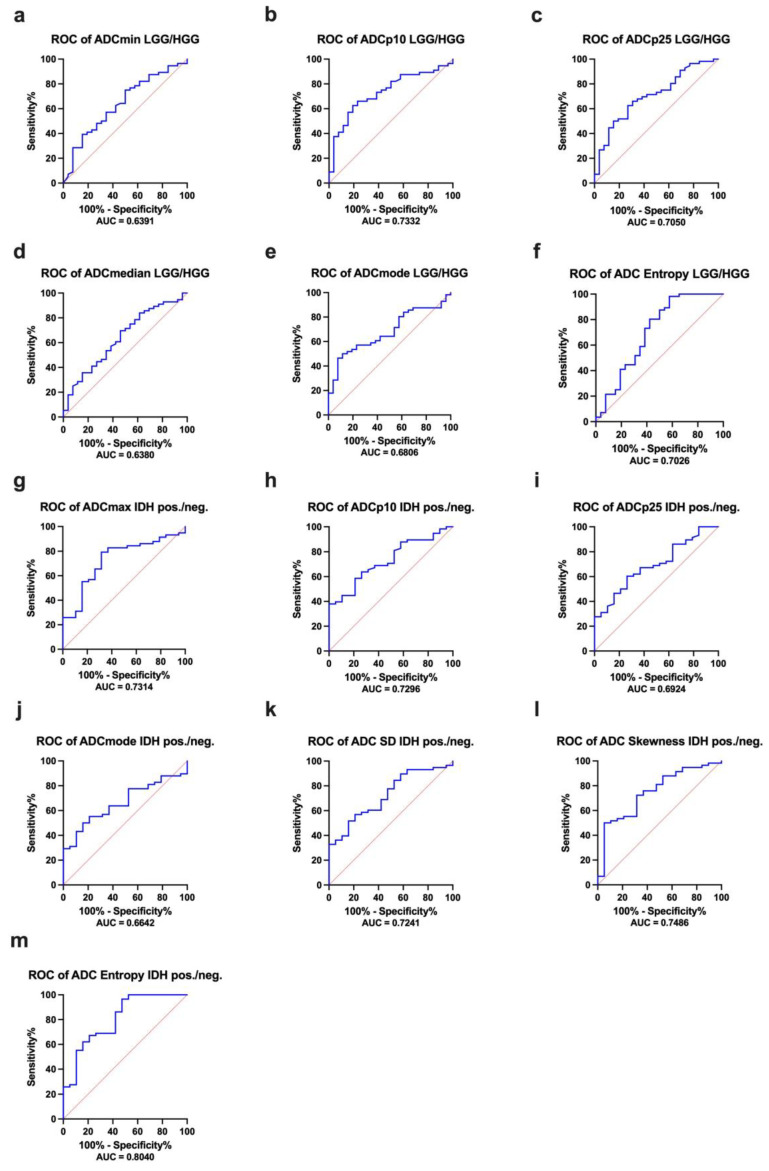
(**a**–**m**) provides the receiver operating characteristics (ROC) curves and the corresponding AUC values of all ADC features that achieved statistical significance comparing LGG to HGG and IDH1-mutated to IDH1-wildtype gliomas.

To evaluate the test performance of the different histogram parameters as possible classifiers in terms of differentiating LGG from HGG and IDH1-mutated gliomas from IDH1-wildtype gliomas, receiver operating characteristic (ROC) analysis and calculation of the corresponding AUC values was performed using those ADC HA features that achieved significance in the comparative statistics. The greatest accuracy concerning the distinction between LGG and HGG was detected for the lowest percentile ADCp10 (AUC = 0.7332, (CI: 0.6214–0.8450), *p* = 0.0007) and concerning the distinction between IDH1-mutated and IDH1-wildtype gliomas for entropy (AUC = 0.8040, (CI: 0.6849–0.9231), *p* < 0.0001). The complete results of the ROC analysis are listed in Table 6; the corresponding ROC curves are displayed in Figure 5.

Finally, Youden’s index was calculated to define the optimal cutoff value for differentiation between LGG and HGG: ADCp10 values ≤ 0.0009805 indicate HGG (sensitivity: 0.81, specificity: 0.63). Furthermore, the calculation of Youden’s index was performed to define the optimal cutoff value for differentiation between IDH wildtype and IDH1 mutant: ADC entropy values ≤ 5.488 indicate an IDH1-wildtype profile (sensitivity: 0.73, specificity: 0.97).

## 4. Discussion

Depending on the underlying tumor grade, gliomas comprise a more or less heterogeneous tissue microarchitecture containing areas of different mitotic activity and cellularity. This tumor heterogeneity is not represented adequately by conventional T1w and T2w images. In the case of HGG, for example, it has been reported that the peritumoral edema—a region that is not characterized by leaky neoplastic vessels and thus parenchymal contrast enhancement—contains aggressive and highly proliferating tumor cell islets [26]. Especially those highly proliferative areas that remain unremarkable in conventional contrast-enhanced MRI sequences are often the origin of local tumor recurrence.

Therefore, presurgical identification of hot spots with increased proliferation—within and outside the morphologically identifiable tumor region—as potential targets for biopsy and open surgery planning is pivotal.

The ADC histogram of a tumor reflects the characteristically altered diffusion profile of proteins inside the extracellular matrix of a neoplasm, which is strongly associated with tumor-cell proliferation and higher tumor grades in a variety of neoplasms, with lower ADC values resulting from higher cellularity and tumor grade [27,28,29,30]. In line with these studies, our results showed significantly decreased first-order ADC histogram parameters; i.e., the minimum values, the lower percentiles as well as the median and mode for HGG, underlining a shift of the ADC continuum towards lower values in association with a higher tumor grade and greater proliferative activity of the respective tumor tissues. Entropy values of the ADC, representing a second-order feature, also exhibited significant differences between LGG and HGG with decreased values in the HGG group. This is a rather unexpected result, since previously published ADC histogram studies suggest ADC entropy as potential imaging marker for tumor heterogeneity with higher values in case of higher tumor grade [10,31,32]. Entropy as a quantity in HA describes the randomness of values, possibly reflecting the degree of ‘chaos’ in the neoplastic microarchitecture. For example, ADC entropy values were reported to be significantly greater in WHO grade II gliomas in comparison to WHO grade I gliomas [10] and exhibited significant changes after treatment of cancer cells with cytoreductive agents [33]. We hypothesize that our finding substantiates an association of ADC entropy with a set of phenotypic features being characteristic of altered tumor behavior, and not an exclusive link between ADC entropy and architectural heterogeneity. In particular context of gliomas, advanced tumor biology has been related to a phenotypic shift from a proneural to mesenchymal subtype [34], which may potentially be reflected by corresponding changes in ADC entropy. However, this hypothesis is of purely theoretical nature and requires further investigations.

In line with the generally accepted concept of increased proliferation in HGG, our study revealed a significantly greater extent of Ki67 immunoreactivity in HGG than in the low-grade neoplasms as shown in Table 2 and exemplarily demonstrated in Figure 1 by the Ki67 immunolabelling sections.

IDH1 and -2 mutations appear early in gliomagenesis in the majority of LGG and secondary HGG [35]. As important prognostic factor, IDH mutation status is a well-established part of the basic histopathological diagnosis in case of gliomas. Tumor entities with mutated IDH genes show better prognosis and favorable individual outcomes in comparison to IDH-wildtype gliomas [36,37,38]. Beside this prognostic relevance, IDH mutation could furthermore play a role as potential therapeutical target in the future and therefore influence the glioma therapy regime [39]. In the present study, we discovered a strong, significant difference (*p* < 0.0001) concerning the entropy values of IDH1-wildtype versus IDH1-mutated tumors, with higher values being present in the latter. These findings are in accordance with a recent imaging study on the ADC histogram analysis of LGG [10]. The entropy of the ADC histogram appears to be a resilient imaging marker reflecting IDH gene mutation status across the different glioma grades, and thus represents a promising biomarker for future studies. Additionally, the present histogram study revealed significantly higher values of ADCmax, ADC SD and skewness as well as lower values of ADCp10, ADCp25 and ADCmodus in the IDH1-wildtype group, which is best explained by the fact that most of the IDH1-wildtype tumors are WHO grade IV entities, together with the abovementioned asymmetrical shift of the ADC histogram profile towards lower values and a possibly broader scattering of ADC values in general.

MGMT represents an important genomic repair mechanism, which has gained importance for the risk stratification of HGG. Silencing its gene expression due to promoter methylation during tumor development enhances the cyto-reductive efficacy of alkylating anti-cancer drugs and has been shown to increase survival rates in the case of GBM [40]. However, the prediction of the MGMT promoter profile via ADC histograms in gliomas showed partly conflicting and ambiguous results [41,42,43,44,45]. Consistent with the negative results of those works, our actual study did not reveal associations of ADC histogram features with the MGMT promoter profile.

Finally, a number of histogram features showed significant, inverse correlations with Ki67 immunolabelling. It is a well-established fact that tumors with a higher tumor grade are associated with increased proliferative activity, accompanied by the higher expression of Ki67 and consecutively increased cellularity, with a reduction in intercellular space and thus extracellular diffusion restriction. Our correlative statistic results suggest a direct and robust correlation between tumor proliferation and tumor ADC histogram profile with a complete shifting of the ADC curve towards lower ADC values, expressed by lower percentiles, extreme values, median and modus, as well as higher skewness. These results are consistent with previous studies investigating different neoplastic intracranial lesions like lymphoma, meningioma and low-grade glioma, which also demonstrated comparable associations of distinct ADC fractions with Ki67 immunolabelling [10,16,17].

Our study has a number of limitations, including its retrospective design using data exclusively from 1.5T scanners with therefore lower signal-to-noise ratios and subsequently ADC maps with reduced spatial information in comparison to 3T scanners. Additionally, the ADC was derived from only two b-values (0 and 1000 s/mm^2^). This may result in biased ADC values, as perfusion effects influence the ADC when using b-values below 200 s/mm^2^. This limitation could be overcome by using DWI with a multiple-b-value approach, which not only makes the calculation of the ADC more accurate, but also provides additional parameters like the pseudo-diffusion coefficient that reflects the perfusion properties of the tissue. Furthermore, related to the preliminary nature of our investigation, the familywise alpha inflation due to serial hypothesis tests in the statistical analysis was not controlled.

## 5. Conclusions

Glioma ADC histogram profiling could aid in the differentiation of LGG and HGG, facilitate the estimation of growth kinetics and allow clinicians to draw preliminary conclusions about the IDH gene profile of the lesion at hand. Therefore, ADC histogram profiling as very straightforward and easily available postprocessing radiomic approach should be implemented in standard presurgical diagnostics to improve the accuracy of diagnosis as well as to help detecting possible hotspots of increased proliferation within the tumor tissue for targeted biopsy.

## Figures and Tables

**Table 1:** ADC histogram features of all included gliomas.

ADC Histogram Features	Mean ± Standard Deviation	Minimum	Maximum
ADC_mean_, ×10^−5^ mm^2^s^−1^	141.11 ± 31.97	66.56	230.91
ADC_min_, ×10^−5^ mm^2^s^−1^	44.00 ± 28.36	0.1	101.30
ADC_max_, ×10^−5^ mm^2^s^−1^	278.35 ± 60.11	107.30	397.80
ADC_p10_, ×10^−5^ mm^2^s^−1^	100.16 ± 20.25	44.50	160.70
ADC_p25_, ×10^−5^ mm^2^s^−1^	115.86 ± 26.49	49.80	203.60
ADC_p75_, ×10^−5^ mm^2^s^−1^	163.91 ± 44.76	75.30	274.00
ADC_p90_, ×10^−5^ mm^2^s^−1^	186.27 ± 48.66	82.79	283.70
ADC_median_, ×10^−5^ mm^2^s^−1^	137.65 ± 35.92	59.85	263.30
ADC_modus_, ×10^−5^ mm^2^s^−1^	137.66 ± 52.57	46.30	277.00
ADC SD, 10^−5^ mm^2^s^−1^	34.87 ± 15.64	8.77	77.46
Kurtosis	4.72 ± 3.65	1.35	23.34
Skewness	0.60 ± 0.95	−2.07	3.80
Entropy	4.87 ± 0.61	3.25	6.19

**Table 2:** ADC histogram features and Ki67 immunolabelling in LGG vs. HGG. Significant results are written in bold letters.

ADC Histogram Features	Low-Grade Glioma Mean ± SD		High-Grade Glioma Mean ± SD		*t*-Test *p*-Values
ADC_mean_, ×10^−5^ mm^2^s^−1^	148.70	32.04	137.60	31.88	0.1446
ADC_min_, ×10^−5^ mm^2^s^−1^	53.75	25.47	39.48	28.96	**0.0433**
ADC_max_, ×10^−5^ mm^2^s^−1^	260.50	59.07	286.60	59.85	0.0604
ADC_p10_, ×10^−5^ mm^2^s^−1^	110.00	15.45	95.59	20.88	**0.0024**
ADC_p25_, ×10^−5^ mm^2^s^−1^	129.30	26.59	109.60	24.47	**0.0014**
ADC_p75_, ×10^−5^ mm^2^s^−1^	167.80	42.13	162.10	46.58	0.4528
ADC_p90_, ×10^−5^ mm^2^s^−1^	185.30	47.07	186.70	50.22	0.8996
ADC_median_, ×10^−5^ mm^2^s^−1^	148.90	36.22	132.40	35.21	**0.0450**
ADC_modus_, ×10^−5^ mm^2^s^−1^	153.20	46.25	130.40	54.60	**0.0083**
ADC SD, 10^−5^ mm^2^s^−1^	30.11	13.84	37.07	16.19	0.0600
Kurtosis	4.23	2.76	4.94	4.03	0.6381
Skewness	0.32	0.89	0.72	0.96	0.0740
Entropy	5.19	0.70	4.73	0.51	**0.0011**
Ki-67	4.71	2.58	25.74	17.82	**<0.0001**

**Table 3:** ADC histogram features in gliomas with and without IDH-1 mutation. Significant results are written in bold letters.

ADC Histogram Features	IDH-1 Mutation Mean ± SD		IDH-1 Wildtype Mean ± SD		*p*-Values
ADC_mean_, ×10^−5^ mm^2^s^−1^	144.60	22.65	138.50	33.03	0.4523
ADC_min_, ×10^−5^ mm^2^s^−1^	54.88	23.27	40.41	29.25	0.0571
ADC_max_, ×10^−5^ mm^2^s^−1^	244.10	51.79	288.40	60.15	**0.0022**
ADC_p10_, ×10^−5^ mm^2^s^−1^	112.50	18.52	95.64	19.89	**0.0017**
ADC_p25_, ×10^−5^ mm^2^s^−1^	127.80	22.62	110.00	24.00	**0.0058**
ADC_p75_, ×10^−5^ mm^2^s^−1^	160.80	26.43	163.40	48.61	0.7899
ADC_p90_, ×10^−5^ mm^2^s^−1^	175.60	28.29	188.50	52.87	0.5170
ADC_median_, ×10^−5^ mm^2^s^−1^	144.60	25.65	133.20	35.53	0.1245
ADC_modus_, ×10^−5^ mm^2^s^−1^	146.60	31.42	132.80	56.84	**0.0320**
ADC SD, ×10^−5^ mm^2^s^−1^	25.78	9.16	37.70	16.19	**0.0030**
Kurtosis	3.82	1.61	5.02	4.14	0.7296
Skewness	0.07	0.82	0.80	0.93	**0.0028**
Entropy	5.42	0.58	4.70	0.54	**<0.0001**

**Table 4:** ADC histogram features in gliomas with and without MGMT promoter methylation.

ADC Histogram Features	MGMT Promoter Methylation Positive Mean ± SD		MGMT Promoter Methylation Negative Mean ± SD		*p*-Values
ADC_mean_, ×10^−5^ mm^2^s^−1^	138.30	31.98	139.60	30.61	0.8605
ADC_min_, ×10^−5^ mm^2^s^−1^	41.96	27.30	40.21	29.64	0.8835
ADC_max_, ×10^−5^ mm^2^s^−1^	271.80	59.61	280.70	66.78	0.4633
ADC_p10_, ×10^−5^ mm^2^s^−1^	99.01	21.24	97.10	20.17	0.3837
ADC_p25_, ×10^−5^ mm^2^s^−1^	112.60	25.37	112.50	24.19	0.9848
ADC_p75_, ×10^−5^ mm^2^s^−1^	161.70	45.81	163.00	44.18	0.6467
ADC_p90_, ×10^−5^ mm^2^s^−1^	184.00	49.19	187.10	48.99	0.7434
ADC_median_, ×10^−5^ mm^2^s^−1^	133.70	34.32	135.90	34.58	0.7767
ADC_modus_, ×10^−5^ mm^2^s^−1^	131.00	50.25	135.50	53.61	0.9329
ADC SD, ×10^−5^ mm^2^s^−1^	34.25	15.51	36.38	16.85	0.5618
Kurtosis	3.92	2.09	4.75	3.60	0.8176
Skewness	0.59	0.71	0.55	0.70	0.8430
Entropy	4.92	0.71	4.87	0.51	0.7397

**Table 5:** Correlations between ADC histogram features and Ki67 immunolabelling in all included gliomas. Significant results are in bold.

ADC Histogram Features	Correlation
ADC_mean_, ×10^−5^ mm^2^s^−1^	r = −0.2044 *p* = 0.0807
ADC_min_, ×10^−5^ mm^2^s^−1^	**r = −0.3107** ***p* = 0.0071**
ADC_max_, ×10^−5^ mm^2^s^−1^	**r = −0.2772** ***p* = 0.0168**
ADC_p10_, ×10^−5^ mm^2^s^−1^	**r = −0.4506** ***p* < 0.0001**
ADC_p25_, ×10^−5^ mm^2^s^−1^	**r = −0.4026** ***p* = 0.0004**
ADC_p75_, ×10^−5^ mm^2^s^−1^	r = −0.1103 *p* = 0.3493
ADC_p90_, ×10^−5^ mm^2^s^−1^	r = −0.0207 *p* = 0.8613
ADC_median_, ×10^−5^ mm^2^s^−1^	**r = −0.2835** ***p* = 0.0144**
ADC_modus_, ×10^−5^ mm^2^s^−1^	**r = −0.2782** ***p* = 0.0164**
ADC SD, ×10^−5^ mm^2^s^−1^	**r = 0.2957** ***p* = 0.0105**
Kurtosis	r = −0.0953 *p* = 0.4192
Skewness	**r = 0.3118** ***p* = 0.0068**
Entropy	r = −0.0974 *p* = 0.4090

**Table 6:** ROC analysis of different ADC histogram features as possible classifiers between LGG and HGG as well as between IDH1-mutated and IDH1-wildtype gliomas.

ROC Analysis LGG versus HGG			
ADC Histogram Features	AUC	Confidence Interval	*p*-Value
ADC_min_, ×10^−5^ mm^2^s^−1^	0.6391	0.5107–0.7674	0.0436
ADC_p10_, ×10^−5^ mm^2^s^−1^	0.7332	0.6214–0.8450	0.0007
ADC_p25_, ×10^−5^ mm^2^s^−1^	0.7050	0.5868–0.8233	0.0029
ADC_median_, ×10^−5^ mm^2^s^−1^	0.6380	0.5099–0.7662	0.0452
ADC_modus_, ×10^−5^ mm^2^s^−1^	0.6806	0.5647–0.7966	0.0088
Entropy	0.7026	0.5671–0.8381	0.0033
**ROC Analysis IDH1-mutated versus IDH1-wildtype gliomas**			
ADC_max_, ×10^−5^ mm^2^s^−1^	0.7314	0.6054–0.8573	0.0026
ADC_p10_, ×10^−5^ mm^2^s^−1^	0.7296	0.6116–0.8476	0.0028
ADC_p25_, ×10^−5^ mm^2^s^−1^	0.6924	0.5653–0.8195	0.0122
ADC_modus_, ×10^−5^ mm^2^s^−1^	0.6642	0.5413–0.7872	0.0325
ADC SD, ×10^−5^ mm^2^s^−1^	0.7241	0.6017–0.8466	0.0035
Skewness	0.7486	0.6235–0.8737	0.0012
Entropy	0.8040	0.6849–0.9231	*p* < 0.0001

## Data Availability

The relevant histogram data are contained in the tables of this article. The primary histogram data on each patient level can be obtained on request from the corresponding author.
